# Evolutionary Repercussions of Avian Culling on Host Resistance and Influenza Virulence

**DOI:** 10.1371/journal.pone.0005503

**Published:** 2009-05-11

**Authors:** Eunha Shim, Alison P. Galvani

**Affiliations:** Department of Epidemiology and Public Health, Yale University School of Medicine, New Haven, Connecticut, United States of America; Hong Kong University, Hong Kong

## Abstract

**Background:**

Keeping pandemic influenza at bay is a global health priority. Of particular concern is the continued spread of the influenza subtype H5N1 in avian populations and the increasing frequency of transmission to humans. To decrease this threat, mass culling is the principal strategy for eradicating influenza in avian populations. Although culling has a crucial short-term epidemiological benefit, evolutionary repercussions on reservoir hosts and on the viral population have not been considered.

**Methods and Findings:**

To explore the epidemiological and evolutionary repercussions of mass avian culling, we combine population genetics and epidemiological influenza dynamics in a mathematical model parameterized by clinical, epidemiological, and poultry data. We model the virulence level of influenza and the selection on a dominant allele that confers resistance against influenza [1], [2] in a poultry population. Our findings indicate that culling impedes the evolution of avian host resistance against influenza. On the pathogen side of the coevolutionary race between pathogen and host, culling selects for heightened virulence and transmissibility of influenza.

**Conclusions:**

Mass culling achieves a short-term benefit at the expense of long-term detriments: a more genetically susceptible host population, ultimately greater mortality, and elevated influenza virulence.

## Introduction

Transmission of the virulent H5N1 influenza A virus from its avian reservoir to humans has become an increasing public health concern, and the virus has spread to 12 countries [3]. The immunological susceptibility of the human population to this novel subtype confers pandemic potential to this virus, particularly if it evolves to become more transmissible among humans, as may be occurring through the evolution of virus subtypes [4], [5]. The case mortality for H5N1 influenza A in humans is 50–70% [6], [7], which is over 25 times higher than the H1N1 Spanish influenza virus that killed an estimated 20 million people in 1918 [8].

To decrease the threat of H5N1 influenza A virus transmission, control policies have to be in place. The immediate aim of a control policy is to reduce the average number of infections produced by an infected individual in a susceptible population (*R_0_*) below 1, thereby curtailing transmission [9]. Mass culling of avian hosts has been the long-standing practice for influenza control within the avian reservoir. Over 100 million chickens were culled in Asia to contain H5N1 between 2004 and 2005 [6]. The economic impact of H5N1 and associated culling for China from February to April 2004 was estimated to exceed 22 billion US dollars [10]. Nonetheless, overall culling has been a successful strategy for containing the emergence of new influenza subtypes, as for example in a 2002. Outbreaks of H5N1 avian influenza have occurred in Hong Kong in chickens and other gallinaceous poultry in 2002 [11]. Infection on a chicken farm was detected during the outbreak, thus control measures including culling and vaccination were implemented. Subsequent virus surveillance showed the outbreaks of highly pathogenic H5N1 avian influenza had been contained [11].

The importance of understanding the potentially counterintuitive evolutionary repercussions of interventions has lead to modeling of vaccination [12] and treatment effects [13] on pathogen virulence. However, the evolutionary impact of interventions on reservoir host populations has been neglected. Disease can impose intense selection for host resistance, particularly when the disease is highly virulent [14], [15], [16], [17], [18], [19]. For instance, myxomatosis resistance evolved rapidly in rabbits upon artificial introduction of the Myxoma virus into Australia and Europe in the 1950s, accompanied by even more rapid virulence evolution [20], [21].

Here we evaluate the evolutionary consequences of mass avian culling on both the host and the pathogen using a mathematical model parameterized by clinical, epidemiological, and poultry data and by modeling selection on a dominant allele that confers resistance against influenza. Our analysis shows that a control strategy that is epidemiologically beneficial can have detrimental evolutionary repercussions in terms of reducing long-term resistance to H5N1, increasing host mortality, and selecting for elevated influenza virulence.

## Methods

### Epidemiological and evolutionary repercussions of mass culling

Pathogens are selected to maximize their basic reproductive number, *R_0_*, defined as the average number of secondary infections produced by an initial infection in a susceptible population [9]. The life history traits of a pathogen, including level of virulence, will be under selection to maximize *R_0_* [12], [22], [23], [24], [25]. Thus, virulence, defined as the rate of disease-mediated host mortality [26], is usually determined by *R_0_* maximization [23], [24], [25]. In a homogeneous population with host resistance, influenza can be driven extinct when the proportion of hosts resistant to infection exceeds the threshold 1−1/*R_0_*. This threshold, although traditionally formulated in terms of immunity, applies whether host resistance is derived from acquired immunity or from genetic resistance. Thus, 1−1/*R_0_* is referred to here as the “resistance threshold”, which may be achieved through a combination of immunity and genetic resistance.

To determine the evolutionary repercussions of avian culling on host resistance and influenza virulence, we model selection on a dominant allele that confers resistance against influenza. For instance, the *Mx* resistance allele, albeit recessive, has been found in a few chicken breeds [2] that were resistant against H5N1 and other influenza strains [1], [27], [28]. In most commercial chicken lines, *Mx* has been rendered dysfunctional by a single amino acid substitution [2].

### Model for selection of dominant allele conferring resistance against influenza

Selection on a dominant allele that confers resistance against influenza [1], [2] in a poultry population is modeled, as is the virulence level of influenza. The initial size of the population is 10 million chickens (*N*), which is equivalent to the average poultry population of a province in Thailand [6]. Individuals are divided into six classes: homozygous resistant *G*, heterozygous resistant *H*, homozygous wild-type susceptible *S*, infection incubation*E*, individuals who are infectious *I*, and recovered with immunity*R*. The interactions among these classes are described by a system of differential equations that couple the population genetics of resistance evolution to an epidemic model of influenza transmission [9], [29]:
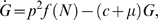
(1)

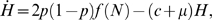
(2)

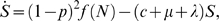
(3)

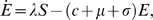
(4)

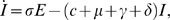
(5)

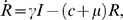
(6)where the Hardy-Weinberg frequency of the resistance allele is 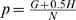 and we assume random mating. The force of infection is 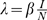 and the density-dependent function of avian reproduction 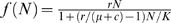. Chickens are culled irrespective of infectious status. Vaccination is modeled as removal of susceptibles to the resistant class (not shown). We assume that a vaccine provides partial protection that results in life-long immunity and that vaccine efficacy is 90% [30]. We compare mass culling to a strategy of selective culling of only symptomatic and infectious chickens, in which removal *c* only occurs from compartment *I*.

Spatially explicit versions of this model are also considered. For this purpose, we assume that breeding occurs within a patch, but transmission can occur between patches at a rate that is 30% of the transmission within a patch. A spatial structure in which all patches are connected is compared with a structure in which a patch is only connected to its nearest neighbors.

The fitness of the avian influenza virus is determined by its rate of propagation through the poultry population. Viral fitness is quantified by *R_0_* [9]:
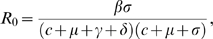
(7)where *β* is the transmission rate, 1/*σ* is the incubation period, *c* is the rate of mass culling, *μ* is the disease-independent host mortality, *γ* is the rate of recovery from infection, and *δ* is the virulence, or rate of disease-mediated mortality (Table 1). Greater host exploitation is likely to simultaneously increase the transmission rate and to decrease host longevity and, hence, the time available for transmission [24], [31], [32], [33]. Thus, we assume a trade-off between persistence of infection (i.e. host survival) and pathogen fecundity (i.e. viral load correlating with transmissibility) [12], [22], [23], [24], [25].

**Table 1:** Table of model parameters employed.

Parameter	Symbol	Value	Reference
Maximal per capita reproduction rate	*r*	Conservative estimate of 0.42 chickens daily	[45], [46]
Carrying capacity	*K*	10 million	[6]
Expected life span	1/*μ*	42 days	[47]
Contact rate per capita	*β*	0.5 contacts daily (derived from 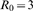 )	[48]
Incubation period	1/*σ*	4 days	[48], [49], [50], [51], [52], [53], [54]
Rate of recovery from infection	1/*γ*	7 days	[4], [53], [54]
Mortality rate	100*δ*/(*γ*+*δ*)	Initially equivalent to an intermediate mortality estimate of 75%	[52]

Elasticity is the percentage rate of change in one variable resulting from a percentage change in another variable. We can show by differentiation of Eq. 7 that the natural log of *R_0_* is maximized when the virulence elasticity of the transmission rate equals the virulence elasticity of the loss of infectiousness,
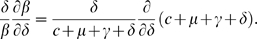
(8)Here, we assume that *β_δ_* is a concave increasing function with an upper bound [34].

## Results

**Figure 1:**
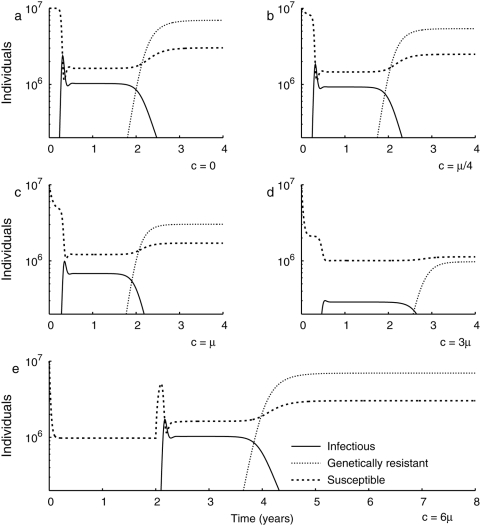
The effect of culling on disease incidence and on the evolution of genetic resistance for continuous culling policies (a–d) and for a policy that discontinues culling after 2 years (e). Increases in the culling rate decrease the transient and influenza incidence (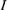) and the eventual level of genetic resistance of the population (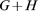). Moderate culling (b,c) may shorten the time for the evolution of genetic resistance compared to no culling (a), but fast culling rates (d) will lengthen the evolution time. Very fast culling rates (e) completely suppress an epidemic, but disease can return when culling is discontinued after 2 years. The simultaneous rebound in the susceptible population corresponds to the abrupt decline in overall mortality.

**Figure 2:**
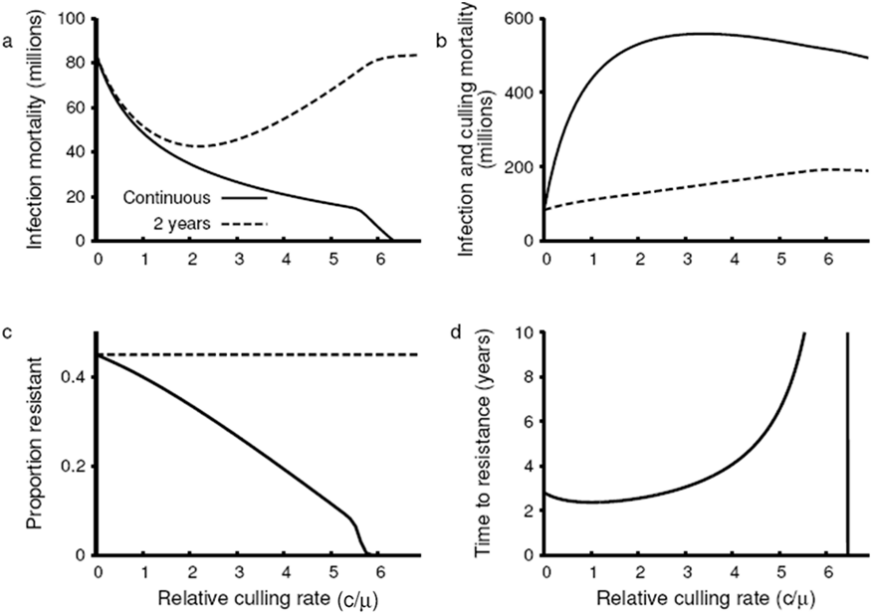
Comparison of outcomes after 10 years for a policy of continuous culling (solid) and a policy where culling is discontinued after 2 years (dashed). (a) The total disease-dependent mortality decreases with the culling rate under continuous culling, suggesting a decrease in the risk of influenza emergence. Very high rates of culling can completely suppress an epidemic, but discontinuation of culling allows the epidemic to resurge. (b) The total mortality due to infection and culling is significantly greater under a continuous policy than under a 2-year policy. Because high rates of continuous culling will ultimately reduce the population size, less mortality will be attributable to culling than would otherwise be expected. (c) The proportion of the population resistant to infection after 10 years decreases with the culling rate under a continuous policy, but the discontinuation of culling allows resistance to eventually reach the same levels obtained in the absence of culling. (d) The time needed for resistance to reach threshold levels (lower as culling increases) is minimized for culling rates that are approximately equal to the background poultry mortality 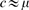. For culling rates above 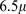, 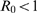. Note that the resistance threshold is not reached within 10 years for culling rates between 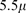 and 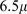.

Culling reduces the average duration of infection and hence the *R_0_* of influenza, thereby decreasing the resistance threshold. Our analysis shows that if culling is sufficiently rapid, it can eliminate influenza without selecting for any host resistance. Our findings indicate that mass culling at a rate that is over six times the rate of background avian mortality is sufficient to drive the *R_0_* of influenza below 1, resulting in the termination of the epidemic (Figure 1e) (at least in the absence of pathogen evolution to an increased *R_0_* [35]). The results are qualitatively robust to variation in *R_0_*, although the rate of culling required increases with *R_0_*. This reduction of *R_0_* and hence of the resistance threshold is the short-term epidemiological benefit of culling in the temporary management of avian influenza. This short-term epidemiological benefit of mass culling is highlighted by the dynamics that arise following the discontinuation of the policy. Such a policy delays the initial progression of the influenza pandemic at the expense of ultimately greater mortality (Figure 2b). As the rate of culling increases, the level of resistance evolved decreases (Figure 2c). After the cessation of culling, the avian population remains vulnerable to H5N1 re-emergence (Figure 1e). Likewise, vaccination reduces the selective pressure acting on host resistance by decreasing transmission although the impacts of vaccination generally last longer than culling. However, unlike culling, vaccinations do not reduce the size of poultry population, making it a more sustainable strategy.

**Figure 3:**
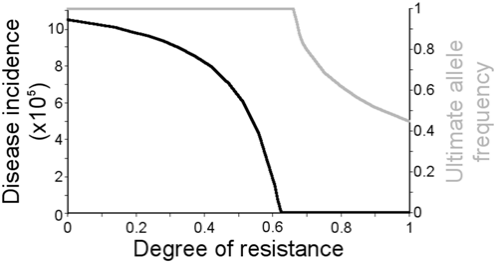
Increase in ultimate allele frequency (top line) and disease incidence (bottom line) with declining degree of resistance conferred by the resistance allele. Degree of resistance is defined as the reduction in the probability of infection compared with a genetically susceptible individual.

The *Mx* allele confers potent resistance against influenza [27]. An allele of lower resistance would increase ultimate allele frequency and disease incidence. The qualitative results of impeded selection due to culling still apply, but as degree of resistance declines, selection will be slower and the frequency of the resistance allele will ultimately be higher (to achieve the same degree of overall fitness), provided that degree of resistance is above 0.66 (Figure 3). Below this threshold, the resistance allele tends to fixation, and population resistance suppresses disease prevalence but is not sufficient to drive influenza extinct. While the *Mx* allele is a single dominant Mendelian resistance allele, the qualitative results will be the same whether resistance is mediated by a single gene or multiple genes, and may be slower or faster depending on epistatic interactions, and the contribution of each allele.

**Figure 4:**
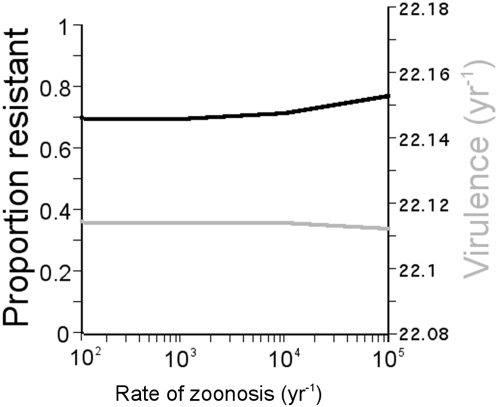
Effect of repeated influenza introductions from a wildfowl reservoir. The effect of repeated emergence of H5N1 on the selection for greater host resistance is negligible until annual rates of emergence reach 10^5^ introductions. The impact of repeated emergence of H5N1 on the rate of influenza evolution towards higher virulence is also negligible for 10^5^ introductions annually. Note that the scale for virulence on the y-axis comprises a very small range.

In the absence of culling, disease prevalence results in a form of density-dependent selection on the host population (Figures 1a, 3). When H5N1 is first introduced into a naive host population, resistance is rare and disease prevalence increases. Simultaneously, the mortality caused by influenza exerts selective pressure on the avian population, leading to a rise in the frequency of the resistance allele. At the resistance allele frequency of 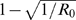, the resistance threshold is reached, and H5N1 is eradicated. Once genetic resistance has evolved, control becomes unnecessary, because the avian population is protected against influenza re-emergence by heritable resistance. The effect of frequent H5N1 emergence from a wildfowl reservoir imposes a negligible to minor additional increase in selection for host resistance (Figure 4).

As the culling rate increases, less host resistance is required to eliminate influenza while culling is maintained. At very high rates of culling, no resistance is required to eradicate disease. However, it generally takes much longer to eliminate influenza as the ratio of culling to background poultry mortality increases from 3 to 6 (Figure 2d). At lower levels of culling, the trade-off between the reduced resistance threshold and less selection for resistance is approximately balanced. Thus, increasing the ratio of culling to background poultry mortality from 1 to 3 has little effect on the rapidity of disease eradication (Figure 2d). Taken together, our analysis shows that there is an optimum level of culling that maximizes the rate at which sufficient resistance is achieved to eradicate disease. This level occurs when the rate of culling is approximately equal to the rate of background mortality. Furthermore, the absolute level of resistance evolved declines as the culling rate increases (Figure 2c), which determines future resistance to H5N1 re-emergence.

Infection mortality is higher for an initial influenza epidemic in the absence of culling (Figure 2a). However, the number of birds killed through a combination of culling and infection is dramatically minimized with decreasing levels of culling (Figure 2b). Thus, there is a trade-off between initial infection (temporary control) and resistance (long-term control).

Antigenic ‘drift’ evolution of influenza was modeled by incorporating waning of immunity over the course of a year. Waning immunity arising from antigenic evolution enhances selection for broad resistance against influenza strains in general, such as that conferred by the *Mx* allele [2]. However, unlike in humans, the mortality rate of commercial chickens is much faster than antigenic drift. Thus, the waning of immunity does not play an important role on the selection of host resistance against influenza in poultry. Even an unrealistically rapid waning of immunity over the course of a month generates at most a 1% greater proportion of resistance after 10 years, provided that culling rate is not sufficient enough to eradicate the disease.

**Figure 5:**
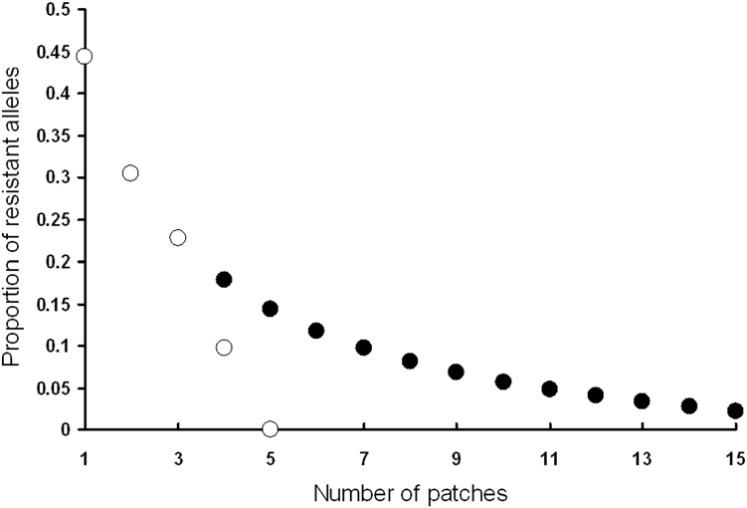
Decline in resistance evolved as the spatial division increases (filled dot: all patches are connected; empty dot: only neighboring patches are connected). Spatial structuring slows the rate of resistance evolution and also reduces the equilibrium level of resistance. Comparing spatial structures of different connectivity shows that the greater the connectivity, the greater the equilibrium level of host resistance. When the density of hosts is decreased, epidemic size is reduced, thus the selection for host resistance is lowered.

Selective culling of only infected and symptomatic chickens is impractical, but is considered for comparative purposes as the opposite extreme of mass culling. Selective culling stunts the evolution of host resistance to a much lesser degree than mass culling (Figure 5). Per chicken culled, selective culling is more efficient at reducing disease than mass culling. However, for a given rate of culling, mass culling is more effective at controlling disease than selective culling although at the expense of more chickens.

Repeated emergence of H5N1 from a wildfowl reservoir does not change the optimal virulence in the chicken population, but could dilute the rate of influenza evolution towards higher virulence. However, this effect is negligible for even 10^5^ H5N1 introductions annually from a wildfowl reservoir into a chicken population of 10 million (Figure 4). The effect on selection for host resistance is also small for realistic rates of emergence from the wild reservoir (Figure 4). As the rate of emergence from the waterfowl reservoir increases, the selection for greater host resistance intensifies. However, this effect is negligible until annual rates of emergence reach 10^5^, a rate that is likely much higher than the actual rate.

**Figure 6:**
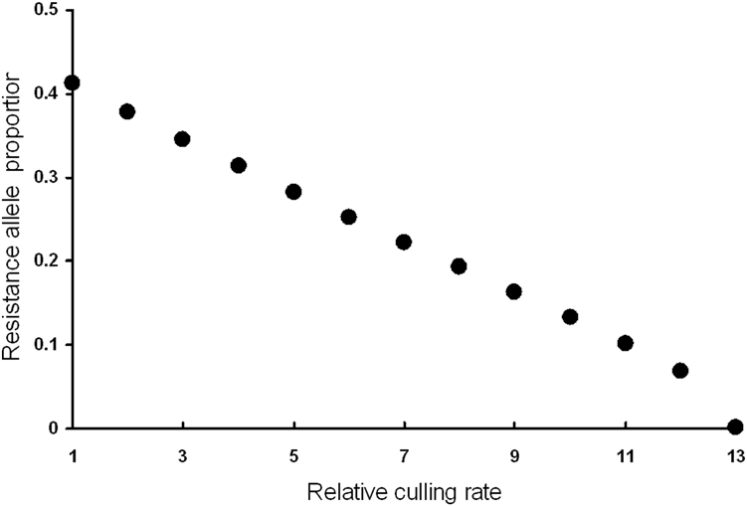
Decrease in the resistance allele evolved as the rate of selective culling increases. Selective culling hinders the evolution of host resistance to a much lesser degree than mass culling.

The principal result that culling reduces selection for host resistance holds with or without spatial structuring of transmission. However, spatial structuring does have dramatic effects on the spread of infection and thus on the evolution of host resistance. Spatial structuring slows the rate at which resistance evolves and also reduces the equilibrium level of resistance (Figure 6) although resistance can rise rapidly within a patch. We also compare a spatial structure in which all patches are connected with a structure in which a patch is only connected to its nearest neighbors. Results indicate that the greater the connectivity, the greater the equilibrium level of host resistance (Figure 6). Similarly, decreasing the density of hosts lowers selection for host resistance by reducing the epidemic.

Culling would reduce selection for any resistance allele whether recessive or dominant. However, recessive alleles are selected so slowly even without culling that the epidemic would have to occur for decades to make an appreciable difference, assuming a low initial frequency of recessive alleles.

**Figure 7:**
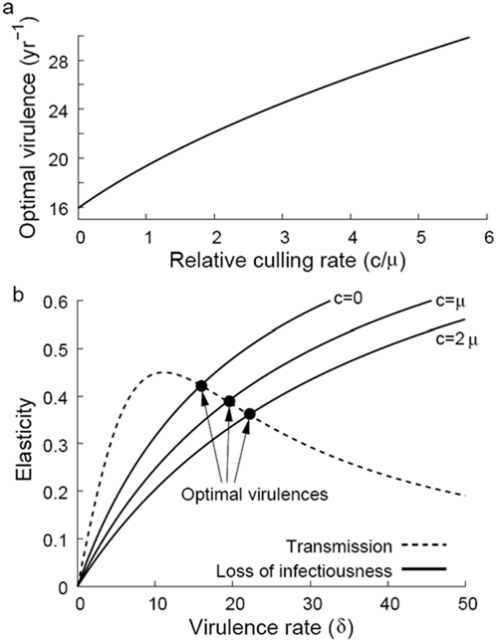
(a) The increase in optimal virulence with culling rate. Virulence is the rate of infection-mediated host mortality. Optimal virulence is a function of culling rate that maximizes overall transmission, *i.e.* 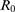. (b) Optimal virulence coincides with the point where the virulence elasticity of the transmission rate is equal to the virulence elasticity of the rate of infectiousness loss. Increases in the culling rate do not affect the elasticity of transmission but always diminish the elasticity of infectiousness loss, so the optimal virulence increases as the culling rate increases. The virulence elasticity of infectiousness loss is plotted for culling rates 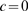, 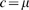, and 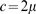.

H5N1 is a rapidly evolving RNA virus, as is exemplified by its already observed evolution. The economic concept of elasticity was used to calculate optimal virulence. Elasticity is the percentage change in one variable resulting from a single percent change in another variable. The virulence elasticity of the transmission rate is a dimensionless and hence generalizable parameter defined as the percentage increase in transmission rate per percentage rise in virulence. Likewise, the virulence elasticity of the infectiousness duration is the percentage increase in infectiousness duration per percentage increase in virulence. Our model shows that optimal virulence occurs where these elasticities are equal (Figure 7a). Movement away from this optimum towards further elevated virulence would generate a greater reduction in the infectiousness duration than in the transmission rate, while movement towards lower virulence would cause a greater decrease in the transmission rate than in the infectiousness duration (Figure 7a). Culling is found to shift the optimum toward higher virulence levels (Figures 7a,b). Thus, increased culling selects for elevated influenza virulence. Even when virulence levels are far from their optimal values (as is likely when there is an influenza emergence event), increased culling is predicted to shift selective pressures toward more virulent and transmissible strains. This result is consistent with previous findings that shorter life expectancy of pathogens can drive the evolution of greater virulence [36], although ultimately virulence could still be less than on initial introduction.

## Discussion

Although mass culling achieves a short-term benefit in disease control, it was found to impede the evolution of host resistance, which is important for long-term success of keeping avian influenza at bay. Culling achieves a short-term benefit at the expense of long-term detriments: a genetically susceptible host population, greater mortality overall, and elevated influenza virulence. Our analysis also indicates that an avian population that evolves genetic resistance in the absence of culling will be resistant to influenza re-emergence, whereas a population that evolves less resistance will require repeated culling. Indeed, despite the mass cullings that have occurred since 1997, H5N1 continues to re-emerge in bird populations.

Avian influenza outbreaks are often initiated by avirulent precursors from wild birds that become virulent in poultry [37], [38], [39], [40]. Ordinarily, while the influenza virus is under selection to transmit as quickly as possible, such selection is constrained because greater host exploitation increases host mortality, thereby terminating transmission. This life history trade-off typically generates intermediate virulence [22], [23], [24], [25]. However, if an avian host is likely to be culled before it dies from influenza, it is optimal for the virus to transmit as much as possible before culling terminates transmission. Consequently, culling selects for elevated virulence. Such selection is exacerbated further by a shortened lifespan for chickens within the poultry industry. The expected lifespan within the poultry industry is only about two months compared to a natural lifespan of several years outside the industry.

Mass culling has been the long-standing practice employed to curtail epidemics of emerging diseases in agricultural and wild animals, such as for rabies and foot-and-mouth disease, in addition to avian influenza. The evolutionary impact of culling has far-reaching implications for conservation, animal welfare, national economies, and human disease emergence. The potential evolutionary repercussions of culling affect both the host and the pathogen. On the host side of the coevolutionary race, culling is found to stunt the evolution of resistance. On the pathogen side, culling selects for heightened virulence. These results suggest that the implementation of mass culling may play a role in the increasingly frequent outbreaks of pathogenic H5N1, H7N3, H9N2 and H7N7 influenza subtypes among poultry [41], [42], [43]. Likewise, H5N1 isolates from domestic birds in China collected between 1999 and 2003 have revealed a pattern of increasing virulence [42], which is consistent with these results. Furthermore, epidemiological studies suggest that the evolution of H5N1 has resulted in mounting transmissibility among humans [4], [5], apparently as H5N1 adapts towards its optimal transmissibility and hence virulence.

Culling has undeniable short-term benefits for disease control, including the possibility of halting a burgeoning epidemic. However, we should be aware of its potential shortcomings. It may be possible to take steps that counter detrimental evolutionary repercussions. Genetic management strategies might facilitate the propagation of resistance by breeding from resistant chickens. Even if mass culling is employed generally, a local strategy, for example, more selective culling, that is ‘evolutionarily friendly’ may lead to a pocket of host resistance that could then be spread more widely through artificial breeding. Reducing the connectivity of poultry farms (e.g. the movement of chickens among farms) and employing vaccination also decrease the selection for host resistance. However, if spatial structuring is maintained or regular vaccination is administered, less host resistance is needed to prevent the spread of an epidemic. Without the evolution of host resistance, these strategies would have to be maintained indefinitely. Nonetheless, whenever an avian influenza outbreak is detected, these strategies may be favorable over culling in that they are likely more sustainable and would involve less morbidity and mortality of both humans and poultry than mass culling. Cost-effectiveness analysis incorporating the epidemiological, agricultural, and evolutionary costs and benefits will help determine the economically optimal strategy. Intermediate rates of culling may be preferable in terms of optimizing epidemiological control, agricultural destruction [44], cost-effectiveness, and evolutionary considerations.

In summary, the results presented here highlight general principles of the impact of culling on influenza evolutionary epidemiology and host evolution. Although our results do not provide a quantitatively precise prescription for intervention, qualitative relationships may have to be considered when devising control strategies. By incorporating evolutionary repercussions of control strategies into our evaluation of management methods and agricultural practices, we may be able to minimize our long-term risk from influenza and other infectious diseases.
